# Role of resveratrol in inhibiting pathological cardiac remodeling

**DOI:** 10.3389/fphar.2022.924473

**Published:** 2022-09-01

**Authors:** Shaowei Fan, Yuanhui Hu, Yaping You, Wenjing Xue, Ruoning Chai, Xuesong Zhang, Xintian Shou, Jingjing Shi

**Affiliations:** ^1^ Department of cardiological medicine, China Academy of Chinese Medical Sciences Guang’anmen Hospital, Beijing, China; ^2^ Graduate School of Beijing University of Chinese Medicine, Beijing, China

**Keywords:** resveratrol, cardiac remodeling, oxidative stress, mitochondrial biogenesis, autophagy, myocardial fibrosis

## Abstract

Cardiovascular disease is a group of diseases with high mortality in clinic, including hypertension, coronary heart disease, cardiomyopathy, heart valve disease, heart failure, to name a few. In the development of cardiovascular diseases, pathological cardiac remodeling is the most common cardiac pathological change, which often becomes a domino to accelerate the deterioration of the disease. Therefore, inhibiting pathological cardiac remodeling may delay the occurrence and development of cardiovascular diseases and provide patients with greater long-term benefits. Resveratrol is a non-flavonoid polyphenol compound. It mainly exists in grapes, berries, peanuts and red wine, and has cardiovascular protective effects, such as anti-oxidation, inhibiting inflammatory reaction, antithrombotic, dilating blood vessels, inhibiting apoptosis and delaying atherosclerosis. At present, the research of resveratrol has made rich progress. This review aims to summarize the possible mechanism of resveratrol against pathological cardiac remodeling, in order to provide some help for the in-depth exploration of the mechanism of inhibiting pathological cardiac remodeling and the development and research of drug targets.

## 1 Introduction

### 1.1 Cardiac remodeling

Cardiac remodeling is a common structural feature in the occurrence and development of a variety of cardiovascular diseases. According to the pathogene, it can be divided into physiological and pathological cardiac remodeling. Physiological cardiac remodeling often occurs in the process of exercise, pregnancy and so on. Pathological cardiac remodeling can include the increase of load caused by many factors, for example, the influence of neurohumoral factors, promoting cardiomyocyte hypertrophy and non-cardiomyocyte proliferation, and finally irreversible pathological process. This article mainly discusses the pathological cardiac remodeling. On the one hand, this change can promote the limited increase of myocardial contractility, increase cardiac output, maintain roughly normal cardiac ejection function, and maintain the normal circulation needs of various organ systems in a certain range; However, on the other hand, when the load increases or the ventricle expands after myocardial infarction, it will produce new stimulation to the ventricle, which will aggravate cardiac remodeling. When the myocardium is hypertrophic, due to the limited blood oxygen supply, it will further affect the myocardial contractility, trigger a variety of neurohumoral reactions, and finally seriously affect the cardiac function, even whole heart enlargement, resulting in serious consequences, such as heart failure and sudden death.

The basis of cardiac remodeling mainly lies in myocardial fibrosis, which is one of the most common pathological changes of heart tissue. It is caused by myocardial interstitial collagen deposition and excessive proliferation of myocardial fibroblasts, and can lead to the impairment of cardiac systolic and diastolic function (Zivarpour et al., 2022). In addition to myocardial fibrosis, myocardial hypertrophy is also a common pathological change. It is mainly characterized by the increase of cardiomyocyte size, the reactivation of fetal gene markers, the disintegration of sarcomere and transcriptional remodeling. At present, the known regulatory factors include heart specific transcription factors, such as myocyte enhancer-binding factor 2 (MEF2) and GATA binding protein 4 (GATA4) (Kroon et al., 2010). Oxidative stress is closely related to myocardial hypertrophy and is known to be related to a variety of signaling pathways, including c-Jun N-terminal kinase (JNK) pathway, mitogen-activated protein kinase (MAPK) cascade pathway and extracellular signal-regulated kinase (ERK)/p38 (Liang et al., 2003; Liang and Molkentin, 2003).

At present, new insights into novel mechanisms of cardiac remodeling still need to be discovered. From the perspective of neurohumoral, the activation of renin-angiotensin-aldosterone system (RAAS) promotes cardiac remodeling and ventricular dilation. At present, angiotensin converting enzyme inhibitor (ACEI) and angiotensin Ⅱ receptor blocker (ARB) are applied in clinic, which has the ability to reduce mortality and improve cardiac ejection function. The application of aldosterone receptor antagonists has been confirmed by relevant studies, which can reduce interstitial fibrosis and interstitial remodeling, inhibit left ventricular hypertrophy, resist myocardial oxidative damage, improve endothelial function, inhibit thrombosis, and improve vascular baroreceptor function (Pitt et al., 2003; Wu et al., 2017). Therefore, heart failure therapy includes inhibiting the cardiac remodeling process. In 2021, the ESC guidelines recommended that the angiotensin receptor-neprilysin inhibitor (ARNI) should be increased in the application range, that is, the dual function of ARB and brain natriuretic peptide (BNP) inhibitors, which can achieve multiple purposes of resisting RAAS activation, inhibiting sympathetic nervous system (SNS) activation, enhancing endogenous BNP level, reducing water and sodium retention, expanding resistance vessels, and delaying ventricular pathological remodeling. Therefore, it is necessary to continue to cooperate with the application of β receptor antagonists and aldosterone receptor antagonists to bring long-term benefits to patients with heart failure as much as possible.

### 1.2 Resveratrol

Resveratrol (3,5,4′- trihydroxytrans stilbene) is a stilbene polyphenol molecule, which exists in more than 70 plants, such as grapes, berries, plums, nuts and peanuts.

As we all know, the imbalance of inflammatory response and oxidation-antioxidant mechanism occupies a considerable position in the occurrence and development of cardiovascular diseases. Resveratrol, as a rich antioxidant, has received considerable attention. It shows a certain therapeutic effect in atherosclerosis, heart failure, myocardial infarction, post infarction arrhythmia and so on.

Resveratrol plays an important role in reducing the risk of atherosclerosis. The key is that resveratrol can reduce the concentrations of total cholesterol (TC), low density lipoprotein cholesterol (LDL-C), very low-density lipoprotein cholesterol (VLDL-C), apolipoprotein B (Apo B), lipoprotein a (Lp a), free fatty acid (FFA) and triglyceride (TG). It also increased the level of high-density lipoprotein cholesterol (HDL-C) (Miura et al., 2003; Penumathsa et al., 2007; Abe et al., 2018). Besides, resveratrol can improve plaque stability, and its anti-atherosclerotic effect is equivalent to that of atorvastatin in animal experiments (Berbée et al., 2013). Moreover, resveratrol can upregulate the expression of cholesterol 7 α- hydroxylase in the liver, which can promote the synthesis of bile acids, further reduce TC and LDL-C, and play the role of multi-channel joint anti-atherosclerosis (Raj et al., 2021a). In addition, Resveratrol can promote vasodilation, inhibit the proliferation of vascular smooth muscle, monocyte adhesion and platelet aggregation through endothelial nitric oxide synthase (eNOS) (Wang et al., 2002). Resveratrol eluting stent, coated balloon catheter or intravascular injection of resveratrol through drug delivery catheter can inhibit intimal hyperplasia, promote re-endothelialization and reduce in stent restenosis (Tolva et al., 2016; Speck et al., 2018; Kamann et al., 2019). In addition, resveratrol can reduce ischemia-reperfusion injury, reduce infarct area and improve cardiac function (Chen et al., 2007; Xi et al., 2009; Lamont et al., 2011; Kazemirad and Kazerani, 2020). Moreover, resveratrol can reduce the release of a variety of bioactive inflammatory factors, such as interleukin-(IL -) 1β、IL-6, tumor necrosis factor-α (TNF- α) and IL-18, the expression of aging related markers (p53, p16 and P19) and nuclear factor-κB (NF-κB) (Feng et al., 2020), so as to play a protective role on cardiovascular system and improve hemodynamics.

Regarding the pharmacokinetics and pharmacodynamics of resveratrol, in fact, the total absorption rate of resveratrol in the intestine is as high as 70%, which can be detected in the serum within 30–60 min after ingestion, but the maximum oral bioavailability is only 20% (Walle et al., 2004). Resveratrol is rapidly transformed by phase II metabolism (Vesely et al., 2021), and is finally eliminated by kidney (Raj et al., 2021a). Resveratrol not only accumulates in tissues, but also rapidly metabolizes into corresponding sulfate and glucuronic acid conjugates (Yu et al., 2002; Böhmdorfer et al., 2017) due to the role of intestinal microorganisms (Bode et al., 2013; Tomás-Barberán et al., 2016) and the specific role of enzymes. According to the pharmacology research, the best time to take resveratrol is in the morning, which helps to improve the bioavailability (Almeida et al., 2009). The safe dose range for resveratrol to function is between 100 and 1,000 mg/day (Vesely et al., 2021). When the dose exceeds 2 mg/day, it shows oxidation, promotes cell apoptosis and aging (Posadino et al., 2019; Shaito et al., 2020), and side effects such as diarrhea, nausea, allergy or headache may occur (Almeida et al., 2009).

Many experimental studies have explored the problems of high biological metabolic rate of resveratrol and difficult to maintain its effective concentration, and have conducted extensive research on its analogues, and some results have been achieved. At present, research has been carried out on new preparations and targeted drug delivery systems of resveratrol, such as cyclodextrin carrier system, multi particle form and colloidal carrier, which can increase the stability of resveratrol *in vivo*, reduce the early degradation of resveratrol in the intestine and liver, and finally achieve the effect of improving the bioavailability (Amri et al., 2012). In addition, nanotechnology has been widely used to improve the solubility, oral bioavailability, stability and controlled release of resveratrol (Singh et al., 2017). Resveratrol nanoparticles have shown significant biological activity *in vitro* and *in vivo*, which may help to improve the application of resveratrol in disease treatment (Annaji et al., 2021).

Currently, the research on resveratrol is more in-depth, especially in the mechanism and related signal pathways. Cardiac remodeling is a necessary hub in the occurrence and development of a variety of cardiovascular diseases. Therefore, it is more and more necessary to strengthen the exploration of the possible mechanism and related signal pathways of resveratrol in cardiac remodeling. Consequently, we reviewed the possible mechanism of resveratrol against cardiac remodeling, in order to provide some help and inspiration for the further exploration of drug target development and new preparation research to inhibit cardiac remodeling.

## 2 Possible relevant mechanisms

In the current clinical research, researchers started to study the role of resveratrol in a wide range of disease types, such as hypertension (Marques et al., 2018; Mazza et al., 2018), heart failure (Gal et al., 2020), coronary heart disease (Hoseini et al., 2019), obesity (de Ligt et al., 2020; Rabbani et al., 2021), diabetes (Seyyedebrahimi et al., 2018; Movahed et al., 2020), chronic obstructive pulmonary disease (COPD) (Beijers et al., 2020), arthritis (Khojah et al., 2018; Marouf et al., 2021), menopausal osteoporosis (Wong et al., 2020), and women related diseases (Bahramrezaie et al., 2019; Thaung Zaw et al., 2021). Comorbidity is also the focus of research. From these diseases, the effects of different doses of resveratrol alone, resveratrol combined with other drugs (or lifestyle adjustment, such as calorie restriction) on blood pressure, heart rate and other cardiac function, vascular function, inflammatory response, glucose and lipid metabolism and other test indicators were discussed, including related transcriptome studies. We have summarized the clinical studies related to cardiovascular diseases of resveratrol in recent 5 years, as shown in Table 1. The results showed that resveratrol played an active role in the prevention and treatment of heart remodeling related diseases.

**TABLE 1:** Clinical trials of resveratrol on cardiovascular diseases in recent 5 years.

No	Year	Resveratrol dose	Test cycle	Disease	Cardiovascular impact	Cardiac outcome	Pathway	Mechanism	Phenotype	References
1	2019	500 mg	4 weeks	T2DM; CHD	IR ↓; HDLC ↑; TC/HDLC↑; TAC ↑; MDA ↓; PPAR-γ ↑; SIRT1 ↑	↓ Endothelial dysfunction; ↓ Macrovascular injury	NF-κB; TNFα-TNFR; SIRT1; NO; ROS	Glycolipid metabolism; Antioxidation	Vascular function; Inflammation; Oxidative stress	Hoseini et al. (2019)
2	2020	330 mg	3×day^−1^	CAD	↑ FMD in CABG surgery patients; ↓ FMD in PCI patients	Affecting hemodynamics	Physical factors; SIRT1; NO	Shear stress; Inflammatory status	Vascular function; Inflammation; Oxidative stress	Diaz et al. (2020)
3	2018	300 mg (trans-resveratrol)	1 week	Hypertension	↑ FMD	↓Endothelial dysfunction (female is better than male); Affecting hemodynamics	NO; ROS	Antioxidation	Vascular function	Marques et al. (2018)
4	2020	100 mg	3 months	HFrEF	Red blood cell aggregation ↓; Microcirculation↑; Tissue perfusion ↑; Oxygen supply ↑; Exercise capacity↑	Improving hemorheology; Improving microcirculation	Physical factors	Antioxidant properties; Modifying plasma proteins	Vascular function	Gal et al. (2020)

T2DM, type 2 diabetes mellitus; CHD, coronary heart disease; IR, insulin resistance; HDLC, HDL-cholesterol; TC, total cholesterol; TAC, total antioxidant capacity; MDA, malondialdehyde; PPAR-γ, peroxisome proliferator-activated receptor γ; SIRT1, sirtuin 1; NF-κB, nuclear transcription factor-κB; TNFα, tumor necrosis factor α; TNFR, tumor necrosis factor receptor; NO, nitrogen monoxide; ROS, reactive oxygen species; CAD, coronary artery disease; FMD, flow-mediated dilation; CABG, coronary artery bypass graft; PCI, percutaneous coronary intervention; HFrEF, heart failure with reduced ejection fraction.

**FIGURE 1:**
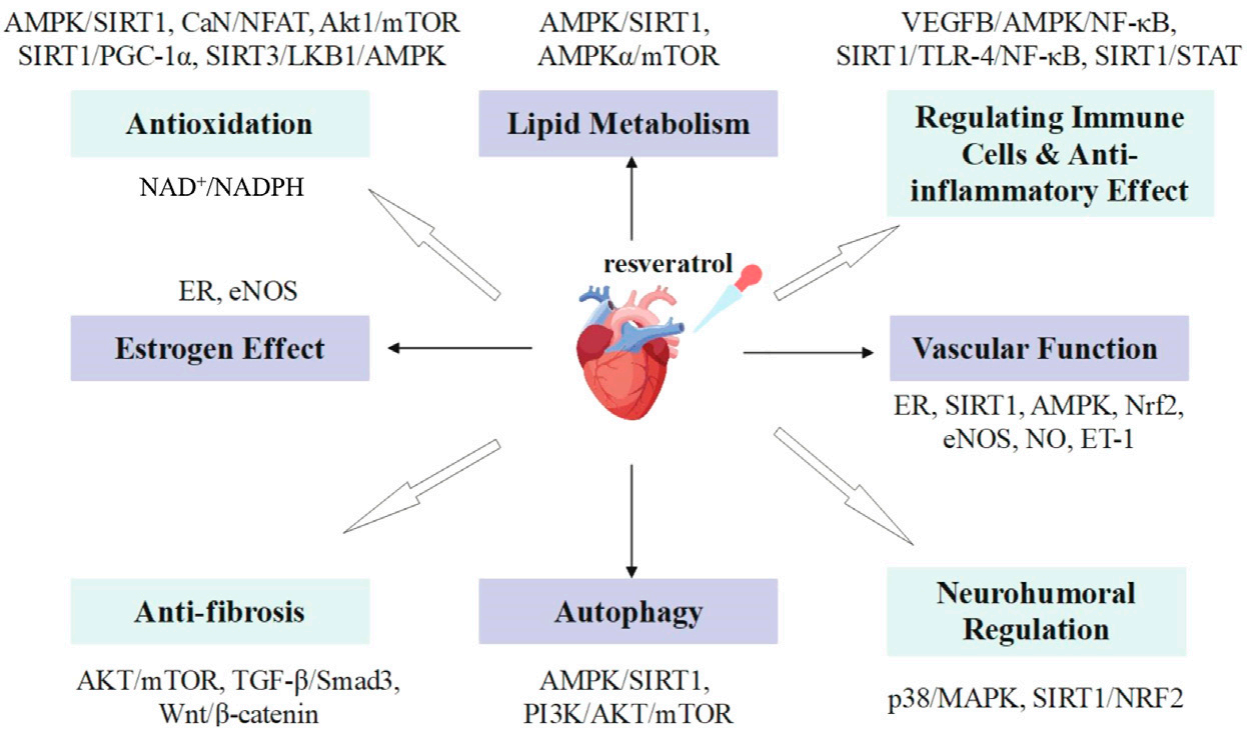
Role of resveratrol in inhibiting cardiac remodeling.

This review will summarize the possible mechanisms of resveratrol inhibiting cardiac remodeling, including reducing oxidative stress and inflammation, myocardial hypertrophy, fibrosis, hypoxia and apoptosis, and promoting autophagy (Figure 1).

### 2.1 Antioxidation

Resveratrol can act as a gene regulator, indirectly scavenging a large number of free radicals, so as to play an antioxidant role. Its function is characterized by multiple pathways: 1) inhibiting the production of reactive oxygen species (ROS) mediated by nicotinamide adenine dinucleotide phosphate (NADPH) oxidase by reducing the expression and activity of oxidase; 2) reducing mitochondrial superoxide production by stimulating mitochondrial biogenesis; 3) upregulation of tetrahydrobiopterin synthase guanosine triohosphte (GTP) cyclohydrolase I and inhibition of uncoupling endothelial nitric oxide (NO) synthase to produce superoxide; 4) upregulating the expression of multiple antioxidant enzymes (Xia et al., 2017).

#### 2.1.1 Upregulation of NAD^+^ level

Silencing information regulator 1 (SIRT1), nicotinamide adenine dinucleotide (NAD^+^) dependent class III histone deacetylase, is an enzyme that consumes NAD^+^ and releases nicotinamide (NAM) and deacetylated adenosine diphosphate (ADP) ribose (Sauve et al., 2006; Cencioni et al., 2015). SIRT1 is an important factor regulating cell survival and longevity. NAD^+^, a signaling molecule, is regulated by a variety of enzymes, such as sirtuins, poly ADP ribose polymerase (PARPs) and some extracellular enzymes, such as CD38. Cyclic adenosine monophosphate (cAMP) can activate protein kinase A (PKA) and SIRT1. And AMP activated protein kinase (AMPK) regulates SIRT1 and intracellular NAD^+^. In addition, AMPK is a negative regulator of protein synthesis and myocardial hypertrophy. It can promote energy production, reduce energy pathway, and inhibit calcineurin (CaN)—nuclear factor of activated T cells (NFAT) pathway, eukaryotic elongation factor (eEF2) and p70S6 kinase (P70S6K). Among them, P70S6K regulates protein translation and extension (Chan et al., 2008). When NAD^+^ is upregulated, it will activate SIRT1 and further promote the deacetylation of peroxisome proliferator-activated receptor γ coactivator 1α (PGC-1α), which in turn regulates more cellular activity. Therefore, NAD^+^ can participate in a series of biochemical reactions, including regulating cell redox state, gene expression, deoxyribonucleic acid (DNA) repair, calcium signal, energy metabolism, mitochondrial biogenesis and circadian rhythm. The heart contains the most abundant NAD^+^, so its metabolic requirements are the highest. In the process of heart failure, the content of myocardial NAD^+^ in cardiomyocytes decreases, due to mitochondrial dysfunction, metabolic remodeling and inflammation (Zhang et al., 2017; Tannous et al., 2021). In the process of aging, the level of NAD^+^ in the heart also decreases, which can lead to cardiac pathological remodeling and dysfunction (Wei et al., 2021). Resveratrol can increase the intracellular NAD^+^ concentration by inhibiting phosphodiesterase (PDE) activity (Park et al., 2012; Li et al., 2019), which helps to inhibit cardiac remodeling. The anti-hypertrophic effect of exogenous NAD^+^ activates SIRT3/liver kinase B1 (LKB1)/AMPK signaling pathway, thereby blocking the hypertrophic effect of mammalian target of rapamycin (mTOR) and AKT serine/threonine kinase 1 (AKT1). In addition, SIRT3 stimulation can reduce ROS level and AKT1 signal and prevent myocardial hypertrophy, which may be one of the mechanisms of resveratrol inhibiting cardiac remodeling (Sundaresan et al., 2009; Pillai et al., 2010; Tannous et al., 2021). Moreover, resveratrol can promote telomerase and can resist the development of cardiac remodeling caused by aging and adverse remodeling after myocardial infarction (Gutlapalli et al., 2020).

#### 2.1.2 Promoting mitochondrial biogenesis

**FIGURE 2:**
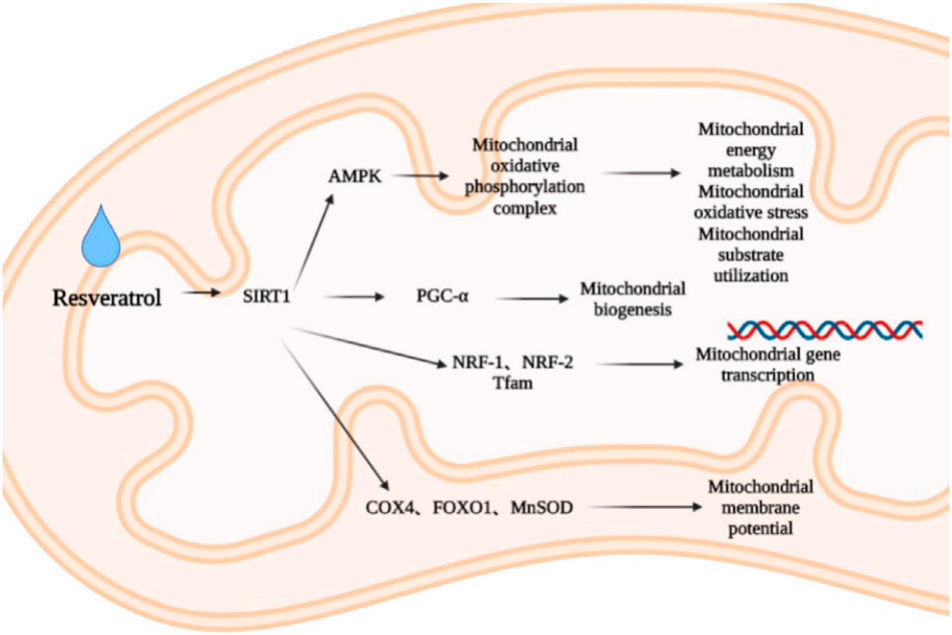
Effect of resveratrol on mitochondrial biogenesis.

Due to the high concentration of mitochondria, cardiomyocytes are vulnerable to oxidative damage such as ischemia or infarction. Therefore, mitochondrial biogenesis is of vital importance to reduce myocardial fibrosis and oxidative stress and maintain the normal structure and function of the heart. Resveratrol is a natural SIRT1 activator (Testai et al., 2020). SIRT1 can improve mitochondrial function and promote mitochondrial biogenesis through deacetylation and activation of PGC-1α (Rodgers et al., 2005; Lagouge et al., 2006). PGC-1α is a major regulator of mitochondrial biogenesis, activating nuclear respiratory factors (NRF) -1 and NRF -2, and inducing gene transcription involved in mitochondrial biogenesis (Scarpulla, 2011). PGC-1α is also activated by AMPK, another important metabolic sensor (Jäger et al., 2007). Resveratrol can downregulate myocardial atrial natriuretic peptide (ANP) level and upregulate PGC-1α, mitochondrial transcription factor (Tfam), NRF-1 and cytochrome c oxidase subunit 4 (COX 4) levels (Biala et al., 2010), and regulate forkhead box protein O1 (FOXO1) and manganese containing superoxide dismutase (MnSOD) levels by activating SIRT1 (Li et al., 2020a), promote the activation of cardiac AMPK, restore the level of mitochondrial oxidative phosphorylation complex (Sung et al., 2015), regulate the energy metabolism, oxidative stress and substrate utilization of myocardial mitochondria (Smeding et al., 2012), inhibiting myocardial fibrosis, improving myocardial hypertrophy and inhibiting cardiac remodeling. Among them, COX is the terminal enzyme of mitochondrial electron transport chain, which is involved in regulating mitochondrial respiration (Lee, 2021). When COX enzyme activity is abnormal, it can affect mitochondrial membrane potential, and then affect the production of adenosine triphosphate (ATP) and ROS, so it can participate in pathological processes such as cardiac remodeling (Sinkler et al., 2017; Lee, 2021) (Figure 2).

#### 2.1.3 Downregulation of superoxide

The application of resveratrol can upregulate the expression level of SIRT1 in rat heart, significantly upregulate the activity of superoxide dismutase (SOD), downregulate the level of myocardial malondialdehyde (MDA) and the expression of 8-oxo-deoxyguanosine (8-OHdG), a marker of DNA damage induced by oxidative stress, in heart, inhibit oxidative stress, apoptosis and fibrosis in cardiomyocytes, reduce the oxidative damage and left ventricular remodeling of aged emphysema rats (Hu et al., 2013). Resveratrol can downregulate MDA, proinflammatory protein TNF- α, and collagen level, a marker of myocardial fibrosis, and can significantly improve cardiac remodeling and reduce systolic dysfunction in rats with myocardial infarction by preventing the decrease of SOD and catalase activities, reducing oxidative stress and the level of cardiac inflammation and fibrosis, and obtain the same therapeutic effect as perindopril (Raj et al., 2016). Nicotine can significantly increase the levels of ROS and 3-nitrotyrosine in rat heart mitochondria, decrease glutathione ratio, and increase left ventricular collagen deposition and indicator transforming growth factor-β (TGF-β), upregulate ANP and BNP, promote cardiomyocyte hypertrophy, fibrosis, oxidative stress, inflammation and left ventricular dysfunction. When isolated rat hearts were subjected to ischemia-reperfusion, left ventricular dysfunction worsened. Resveratrol can reverse the changes of the above indicators, significantly reduce the adverse cardiac remodeling caused by nicotine and the aggravation of ventricular dysfunction after ischemia. It shows that resveratrol targeting mitochondrial ROS can partially reduce cardiac remodeling and dysfunction induced by nicotine (Ramalingam et al., 2021).

#### 2.1.4 Upregulation of antioxidant enzymes

Resveratrol can activate SIRT1/PGC-1 α. Then, it interacts with the transcription factor NRF2, upregulates the expression of heme oxygenase 1 (HO-1) and SOD1, promotes the production of antioxidant enzymes, and plays the role of anti-apoptosis and antioxidant, downregulates the apoptosis of cardiomyocytes caused by myocardial ischemia-reperfusion, and inhibits adverse cardiac remodeling (Wang et al., 2021). Furthermore, it can activate NRF2 signaling pathway and upregulate the expression of antioxidant enzymes. In addition, resveratrol can indirectly increase the activity of SIRT1 by enhancing the combination of SIRT1 and laminin A [LNA, endogenous SIRT1 activator (Liu et al., 2012)], and finally plays a regulatory role on vascular function and antioxidant effect.

### 2.2 Regulating lipid metabolism

Lipid metabolism plays a significant role, which can affect the normal function of cells and systems. Excessive lipid will lead to excessive fatty acid oxidation, inhibit the utilization of glucose through Randle cycle (Goodpaster and Sparks, 2017), causing mitochondrial dysfunction, promoting the accumulation of ROS (Anderson et al., 2009; Boudina and Abel, 2010), inducing cardiotoxicity and inducing cardiac insufficiency. But its specific mechanism is still unclear (Hu et al., 2020). Peroxisome proliferator activated receptor (PPAR) has three subtypes: PPARγ, PPARδ and PPARα (Rosen and Spiegelman, 2001). PPARα and PPARγ participate in regulating lipid and glucose metabolism, provide anti-inflammatory and increase insulin sensitivity (Gervois et al., 2004; Evans et al., 2005). PPARα regulates lipid metabolism and inhibit myocardial fibrosis and cardiac remodeling induced by pressure overload. Resveratrol directly binds PPARγ and PPARα to inhibit PPAR transcription (Calleri et al., 2014). Resveratrol inhibits the differentiation of 3T3-L1 adipocytes by activating AMPK/SIRT1 (Chen et al., 2011), and regulates AMPKα/mTOR pathway mediating autophagy and affecting lipid metabolism (Zhao and Fan, 2020). Resveratrol can downregulate TGF-β and Bax, participate in improving the cardiac histomorphological changes caused by malignant hypertension, inhibit lipid peroxidation, significantly improve the oxidation state and NO release, so as to prevent myocardial hypertrophy and apoptosis and play a therapeutic and preventive role in cardiac remodeling (Grujić-Milanović et al., 2021). Low dose resveratrol treatment can restore the fatty acid oxidation level of myocardial infarction rats, significantly improve cardiac energy metabolism, significantly inhibit cytochrome P450 1B1 (CYP1B1) and cardiotoxic hydroxyeicosatetraenoic acid (HETE) metabolites induced by myocardial infarction rats, significantly improve ejection fraction and inhibit cardiac remodeling (Matsumura et al., 2018a). In addition, resveratrol can also improve perivascular adipose tissue and play a cardiovascular protective role (Zivarpour et al., 2022). Resveratrol helps to optimize the oxidative utilization of fatty acids in cardiomyocytes, improve cardiac function, reduce left ventricular wall stress, prevent cardiac remodeling, and prevent insulin resistance (IR) (Dolinsky et al., 2012).

### 2.3 Regulating immune cells and anti-inflammatory effects

Immune cells participate in cardiac remodeling, which is mostly related to myocardial ischemia, infarction and other traumatic factors. The release of related inflammatory factors may aggravate cardiomyocyte death and injury area, but stimulating angiogenesis may help eliminate inflammatory reaction and accelerate the recovery of the heart (Frangogiannis, 2014; Kologrivova et al., 2021). A variety of cell populations of the immune system have been found to have both pro-inflammatory and anti-inflammatory effects, which is similar to the coagulation and fibrinolysis of human vascular injury. It can not only ensure the rapid and effective repair of the damaged parts, maintain the cardiac morphology and basic function, prevent further deterioration, but also inhibit the continuous progress of inflammatory reaction, promote the recovery of myocardial homeostasis and ensure the recovery of cardiac function (Isobe et al., 2012). In the process of cardiac remodeling, macrophages play an essential role in the inflammatory response (Pullen et al., 2020).

Resveratrol regulates immune cells mainly by activating SIRT1. SIRT1 is a deacetylase that maintains the tolerance of peripheral T cells. When resveratrol binds to SIRT1, on one hand, the conformation of SIRT1 changes and increases the ability to bind to the substrate (Borra et al., 2005). SIRT1 has many substrates, among which p65 (also called Homo sapiens v-rel avian reticuloendotheliosis viral oncogene homolog A, RelA) is a NF-κB member, which can promote leukocyte activation and inflammatory cytokine expression, such as TNF-α, IL-1β, IL-6, monocyte chemoattractant protein 1 (MCP-1), matrix metallopeptidase (MMP) 1 and MMP3, and COX-2 (Yamamoto and Gaynor, 2001). On the other hand, resveratrol activates SIRT1, inhibits RelA acetylation and downregulates the expression level of inflammatory factors, so as to play a role in inhibiting inflammation (Saqib et al., 2018).

#### 2.3.1 Macrophage

**FIGURE 3:**
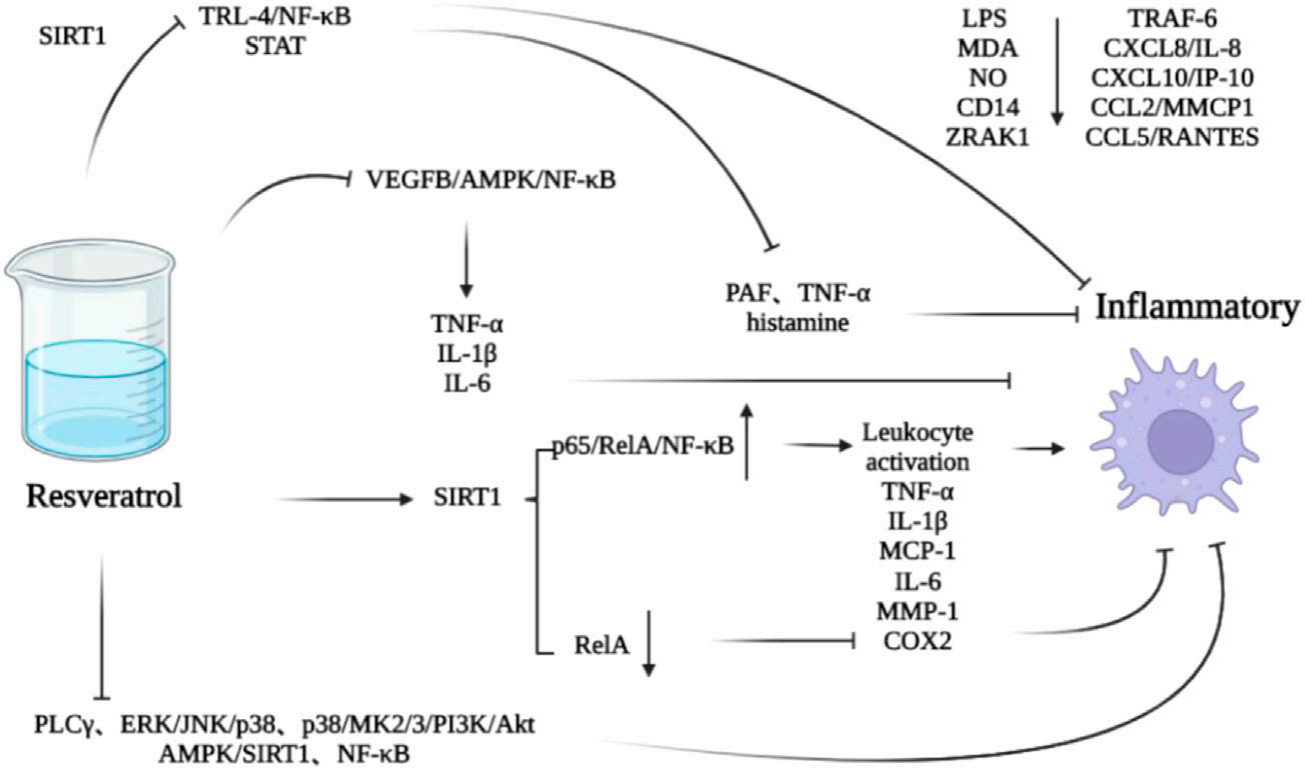
Effect of resveratrol on macrophage mediated inflammation.

In the normal mature heart, a small amount of macrophages from embryonic yolk sac cells are involved in maintaining the homeostasis of atrioventricular conduction (Hulsmans et al., 2017). After injury, macrophages derived from monocytes replace them (Epelman et al., 2014; Heidt et al., 2014), aggregate and activate (Frangogiannis et al., 1998a; Dewald et al., 2005), and participate in matrix remodeling and myocardial fibrosis by secreting fibrosis-related cytokines and growth factors, so as to promote cardiac remodeling (Frangogiannis, 2019). Macrophages have two types, M1 and M2. The former produces TNF-α, IL-1β and IL-6, which are involved in the process of inflammatory response (Lamkin et al., 2016; Hamidzadeh et al., 2017; Hu et al., 2019), and the latter can produce IL-10 and TGF- β1 and other anti-inflammatory cytokines to inhibit inflammation and promote tissue repair (Nahrendorf et al., 2007). Inflammation is associated with M2 polarization of alternately activated macrophages (Tan et al., 2016; Chang et al., 2020). M2 macrophages have four subtypes: M2a can participate in tissue repair (Koh and DiPietro, 2011); M2b is involved in immune regulation (Hagemann et al., 2009); M2c is involved in clearing apoptotic cells (Zizzo et al., 2012); M2d is involved in angiogenesis (Colin et al., 2014). Resveratrol can inhibit M1 polarization of macrophages and upregulate the level of M2 marker by vascular endothelial growth factor B (VEGFB)/AMPK/NF- κB pathway (Li et al., 2020b), so as to play an anti-inflammatory role and inhibit cardiac remodeling. And it can interfere with toll like receptor (TLR)—4/NF-κB and signal transducer and activator of transcription (STAT) through SIRT1 downregulating the level of proinflammatory factors secreted by macrophages/mast cells, such as platelet activating factor (PAF) and TNF- α and histamine (Capiralla et al., 2012). In addition, it can also play an anti-inflammatory role by downregulating MDA, NO, CD14, interleukin 1 receptor associated kinase 1 (IRAK1), TNF receptor associated factor 6 (TRAF6) and chemokines mediated by lipopolysaccharide (LPS), such as chemokine (C-X-C motif) ligand 8 (CXCL8)/IL-8, CXCL10/γ-interferoninducible protein10 (IP-10), chemokine (C-C motif) ligand 2 (CCL2)/MCP1 and CCL5/regulated on activation normal T cell expressed and secreted (RANTES) (Sebai et al., 2010; Jakus et al., 2013; Arango Duque and Descoteaux, 2014). Resveratrol can also reduce the loss of cardiac stem/progenitor cell (CSPC), protect the function of mature cardiac cells, inhibit inflammatory reaction, and inhibit the pathological cardiac remodeling induced by diabetes (Delucchi et al., 2012) (Figure 3).

#### 2.3.2 Mast cells

Mature myocardium also contains a small number of mast cells. Mast cells are stimulated by complement, reactive oxygen species, adenosine or cytokines to degranulate and release mediators that promote the activation of fibroblasts, such as TNF- α (Frangogiannis et al., 1998a), TGF- β (Shiota et al., 2003), IL-4 (Kanellakis et al., 2012) and platelet-derived growth factor (PDGF) (Nazari et al., 2016). Moreover, mast cells produce two specific proteases: tryptase and chymotrypsin (de Almeida et al., 2002). Chymotrypsin can receive the signal of angiotensin converting enzyme (ACE), produce angiotensin Ⅱ (Urata et al., 1990) and activate MMPs (Stewart et al., 2003), so as to promote myocardial fibrosis and cardiac remodeling. Tryptase promotes the process of fibrosis through two pathways: protease activated receptor (PAR)—2 and ERK/MAPK signal (McLarty et al., 2011). Through the study of infarcted myocardium, it is found that the upregulated stem cell factor (SCF) (Frangogiannis et al., 1998b) and the increase of pressure load may promote mast cell aggregation, induce myocardial fibrosis and participate in cardiac remodeling (Levick et al., 2009). However, some studies have reported that mast cells may have anti-fibrosis effect in some cases (Joseph et al., 2005). Therefore, the mechanism and influencing factors of their anti-fibrosis effect still need to be further studied. When mast cells are stimulated by IL-33/growth stimulation expressed gene 2 (ST2), they induce inflammatory response through NF-κB pathway and MAPK-activated protein kinase (MK) 2/3/phosphatidylinositol-3 kinase (PI3K)/AKT pathway downstream of p38, while resveratrol can block these pathways and inhibit the inflammatory response process participated by mast cells in a dose-dependent manner (Nakajima et al., 2019; Xu et al., 2020). Resveratrol can inhibit mast cell degranulation, prostaglandin D2 synthesis and the production of some cytokines through phospholipase C (PLC) γ, ERK/JNK/p38, AMPK/SIRT1 and NF-κB (Han et al., 2013; Li et al., 2017a).

#### 2.3.3 T lymphocyte

When T lymphocytes are activated in ischemia, infarction and increased pressure load, they may induce adhesion molecules through chemokines (Dobaczewski et al., 2010a) and neurohumoral pathways (Amador et al., 2014; Salvador et al., 2016; Li et al., 2017b), infiltrate and recruit, participate in myocardial fibrosis and induce cardiac remodeling (Nevers et al., 2015; Abdullah and Jin, 2018). When the pressure load is too large, Th1 activation will induce fibroblasts to synthesize TGF- β, thus fibroblasts transform into myofibroblasts (Nevers et al., 2017). Both Th2 and Th17 are upregulated in fibrotic myocardium (Cieslik et al., 2011; Duerrschmid et al., 2015), but the specific mechanism needs further study. Regulatory T cells (Tregs) are a kind of T cell subsets, which have the effect of anti-fibrosis (Kvakan et al., 2009; Kanellakis et al., 2011), but also have the ability to secrete fibrogenic factors, such as TGF- β. This characteristic may be related to the ambient signal, which has not been revealed yet. Resveratrol increases the amount and activity of Tregs through the activation of aryl hydrocarbon receptors (Nevers et al., 2017). Resveratrol inhibits T lymphocyte activation through the pathway of SIRT1/T cells c-Jun acetylation (Malaguarnera, 2019).

### 2.4 Improving vascular function

Resveratrol protects vascular function through a variety of mechanisms, including inhibiting oxidative stress and inflammatory response, promoting NO synthesis, inhibiting the proliferation of vascular smooth muscle cells (VSMC), promoting autophagy and so on (Breuss et al., 2019). Estrogen receptor (ER), SIRT1, AMPK and NRF2 all play an important role in regulating vascular function (Li et al., 2012). SIRT1, AMP activated protein kinase and ER are the main molecules mediating the vascular effect of resveratrol, while NRF2 is the indirect target (Li et al., 2019). SIRT1 activator resveratrol can maintain the integrity of endothelial mitochondria and the stability of endothelial internal environment, and reduce endothelial dysfunction (Kim et al., 2015). It can upregulate the expression of eNOS, upregulate the activity of eNOS, inhibit eNOS uncoupling, promote the production of NO in endothelial cells, inhibit the enhancement of oxidative stress induced by eNOS uncoupling, expand blood vessels, improve smooth muscle cell proliferation, vascular remodeling and arterial stiffness, and inhibit the progress of cardiac remodeling (Förstermann and Münzel, 2006; Li and Förstermann, 2013). Resveratrol can also inhibit the phosphorylation of ERK-1/2, upregulate the activity of NADPH oxidase, and inhibit the oxidative stress response of endothelial cells and smooth muscle cells by blocking the synthesis of endothelin-1 (ET-1) (Li et al., 2019). In addition, resveratrol can inhibit the infiltration of immune cells into the vascular wall, so as to play a protective role on vascular function and blood pressure. It also alleviates the vascular lesions and inhibits the decrease of arterial compliance in spontaneously hypertensive heart failure rats (Lee et al., 2017), finally regulating vascular function.

### 2.5 Improving neurohumoral regulation

**FIGURE 4:**
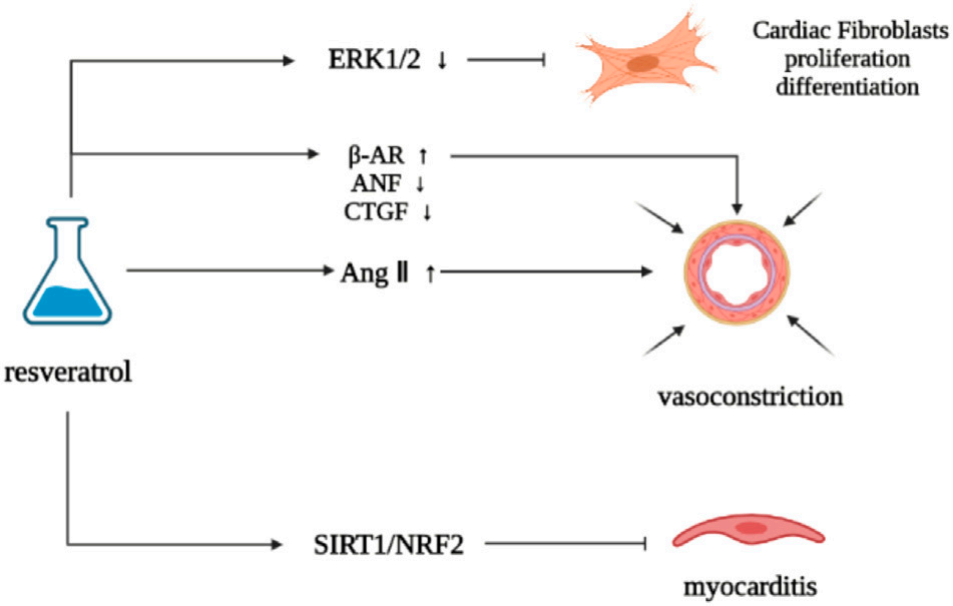
Effect of resveratrol on neurohumoral regulation.

Resveratrol can inhibit cardiac remodeling induced by angiotensin II (Ang II), and has obvious antihypertensive effect and improve myocardial hypertrophy (Biala et al., 2010). It can upregulate the density of β adrenoceptor in myocardial infarction rats to a level similar to that in normal rats, reduce the increase of atrial natriuretic factor (ANF) and connective tissue growth factor (CTGF) expression induced by myocardial infarction, retain myocardial contraction reserve, and inhibit cardiac remodeling to a certain extent, but will not change the changes of sarcoplasmic reticulum Ca^2+^—ATPase 2 and TGFβ1 expression induced by myocardial infarction (Burstein et al., 2007). The use of resveratrol can help restore the normal state of p38/MAPK activation of cardiomyocytes, downregulate the cardiac hypertrophy induced by Ang II, inhibit cardiac remodeling, and provide a guarantee for the clinical application of adriamycin and other drugs (Matsumura et al., 2018b). Stress cardiomyopathy is characterized by β- adrenergic receptor (β- AR) inflammatory body activation, cytokine cascade, M1 macrophage infiltration and pathological cardiac remodeling caused by over activation (Xiao et al., 2018). Resveratrol can target SIRT1/NRF2 signaling pathway, reduce myocarditis and inhibit pathological cardiac remodeling (Nie et al., 2020). Resveratrol can downregulate ERK 1/2 induced by basal cells and Ang Ⅱ, and inhibit cardiac fibroblasts (CFs) growth, proliferation and differentiation into myofibroblast phenotype induced by Ang II and TGF-β, which have concentration-dependent characteristics, and resveratrol plays a vital role in inhibiting myocardial fibrosis and cardiac remodeling (Olson et al., 2005) (Figure 4).

### 2.6 Inducing autophagy

**FIGURE 5:**
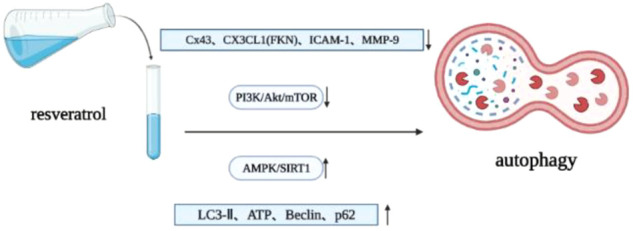
Resveratrol regulates autophagy.

Autophagy is a strictly regulated intracellular catabolic process. With the help of lysosomes, autophagy can degrade misfolded proteins and damaged organelles, clear aging cells, maintain energy balance, and ensure cell survival, homeostasis and function (Glick et al., 2010; Yang and Klionsky, 2010; Singh and Cuervo, 2011). Myocardial autophagy disorder is an important cause of various heart diseases, including pathological cardiac remodeling, myocardial infarction, heart failure and various types of cardiomyopathy (Munasinghe et al., 2016; Saito et al., 2016; Shirakabe et al., 2016; Zhang and Ren, 2016). Autophagy has dual effects. Excessive autophagy can also cause heart damage, but an appropriate level of autophagy is beneficial to the heart. Therefore, autophagy is a key target for the treatment and prevention of heart disease. Resveratrol can upregulate autophagy, reduce collagen deposition, protect myocardium and reduce or reverse cardiac remodeling by activating AMPK/SIRT1 pathway (Pulakat and Chen, 2020; Zhou et al., 2021). When cells (including pulmonary arterial smooth muscle) are activated by extracellular mitogenic factors such as PDGF, epidermal growth factor (EGF), fibroblast growth factor (FGF), insulin-like growth factor (IGF) and ET-1, AKT/mTOR signaling pathway plays an important role in regulating cell proliferation, migration, apoptosis and protein synthesis, most of which are related to subsequent mTOR activation. MTOR is an important downstream signal protein (Babicheva et al., 2021). Resveratrol can upregulate autophagy, downregulate oxidative stress and apoptosis through PI3K/AKT/mTOR pathway, significantly improve the ejection fraction of chronic intermittent hypoxia (CIH) rats, and inhibit myocardial hypertrophy and cardiac remodeling (Guan et al., 2019). In addition, resveratrol can protect the heart by upregulating AMPK and downregulating mTOR. In terms of protein effects, resveratrol upregulates the content of myocardial microtubule associated protein-1 light chain 3-II (LC3-II), ATP and autophagy vacuoles (Kanamori et al., 2013), downregulates the expression level of connexin 43 (Cx43), reduces the ratio of LC3-II/LC3-I, upregulates the expression of Beclin-1 and p62, and promotes autophagy (Wang et al., 2017). The levels of fractalkine (FKN, also known as CX3XL1) in myocardium and serum of mice or patients with chronic heart failure increase, and FKN can induce the upregulation of hypertrophy and heart failure related genes (Husberg et al., 2008). Resveratrol can inhibit the activation of FKN on the expression of ANP in cardiomyocytes, the expression of intercellular adhesion molecule-1 (ICAM-1) in microvascular endothelial cells and the expression of MMP-9 and procollagen in fibroblasts, upregulating the autophagy of cardiomyocytes and inhibiting cardiac remodeling (Xuan et al., 2012) (Figure 5).

### 2.7 Myocardial fibrosis inhibition

**FIGURE 6:**
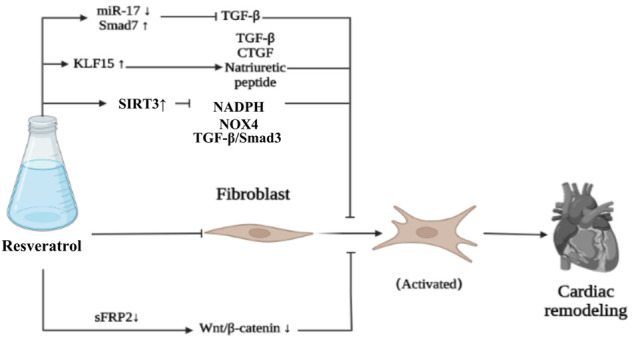
Resveratrol inhibits fibrosis.

Transforming growth factor-β1 (TGF-β1) is one of the most influential fibrogenic factor. Resveratrol can inhibit the proliferation of CFs and collagen secretion of cardiac fibroblasts induced by TGF-β1 by downregulating miR-17 and upregulating Smad7, so as to inhibit cardiac remodeling (Zhang et al., 2018). Krüppel like factor 15 (KLF15) is a transcriptional regulator, which can resist cardiac remodeling and has been proved to regulate TGF-β1 (Zhang et al., 2018), CTGF (Wang et al., 2008) and natriuretic peptides (Fisch et al., 2007; Leenders et al., 2012). Resveratrol can upregulate the expression of KLF15, which may downregulate TGF- β1 and NOx NADPH oxidase (NOX4), upregulate glutamate-cysteine ligase catalytic (GCLC), and finally inhibit cardiac remodeling caused by chronic myocardial infarction (Rogers and Otis, 2017). FGF2 plays an important role in cardiac remodeling in diabetic patients with myocardial hypertrophy and fibrosis. *In vivo* experiments showed that resveratrol could downregulate the expression of Glypican-1, Syndecan-4, FGF2, PPARγ and AMPK in diabetic rats, and alleviate left ventricular diastolic dysfunction, in which FGF2 and heparan sulfate proteoglycan (HSPGs: Glypican-1 and Syndecan-4) were involved in cardiac remodeling (Strunz et al., 2017). In addition, resveratrol can reverse insulin resistance, inhibit myocardial fibrosis and protect the heart by up-regulating mitochondrial enzyme activity, inhibiting the production of reactive oxygen species, and regulating glucose metabolic enzymes, such as glycogen synthase kinase-3β (GSK-3β) and aldose reductase (AR), so as to play a therapeutic role in diabetes cardiomyopathy (Ahmad and Hoda, 2020; Bhagani et al., 2020). Resveratrol can downregulate immune proteasome activity and catalytic subunit (β-1i, β-2i and β-5i) induced by transverse aortic constriction (TAC) , inhibit phosphate and tension homology deleted on chromosome ten (PTEN) degradation, downregulate AKT/mTOR expression, and promote AMPK signal activation, so as to significantly inhibit myocardial hypertrophy, fibrosis and apoptosis and improve cardiac function (Chen et al., 2019). As we all know, SIRT (SIRT1 –7) belongs to class III histone deacetylase, and its activity is closely related to NAD^+^. Among the seven SIRTs, SIRT3 is the only SIRT analogue and has been proved to be positively correlated with human life span (Bellizzi et al., 2005). Upregulation of SIRT3 has been shown to protect cardiomyocytes from genotoxicity and oxidative stress (Sundaresan et al., 2008). Resveratrol can upregulate the level of SIRT3 in cardiac fibroblasts, inhibit the transformation of fibroblasts into myoblasts, inhibit collagen deposition, inhibit myocardial fibrosis and cardiac remodeling, and improve cardiac function by downregulating TGF-β/Smad3 pathway (Chen et al., 2015; Zhang et al., 2021). The use of resveratrol can significantly improve the cardiac remodeling ability of mesenchymal stem cells (MSCs) and inhibit cardiac remodeling by downregulating secreted frizzled-related protein2 (sFRP2) mediated myocardial fibrosis and downstream Wnt/β-catenin pathway (ShamsEldeen et al., 2019). In addition, resveratrol can downregulate the gene expression of disease markers of hypertrophy and extracellular matrix remodeling, and downregulate the expression of collagen type 1 (COL1), collagen type 3 (COL3), MMP2 and tissue inhibitor of metalloproteinase (TIMP-1, -2, -3 and -4), upregulate myocardial insulin sensitivity to promote glucose metabolism, upregulate cardiac AMPK activity, restore mitochondrial oxidative phosphorylation protein level and cardiac glucose metabolism, so as to inhibit myocardial fibrosis in various ways, reduce cardiac remodeling and improve cardiac function (Sung et al., 2015; Chen et al., 2021) (Figure 6).

### 2.8 Estrogen like effects

Resveratrol is a kind of polyphenol, belonging to phytoestrogen, and has homology with estrogen in structure and function. Therefore, resveratrol can play a role in human body, activate ER (Gehm et al., 1997), and have a weak role of endoplasmic reticulum activator/antagonist (Bowers et al., 2000; Kubo et al., 2003; Novakovic et al., 2022). Different regulation of estrogen and its receptor can affect cardiac remodeling. Currently known ERs include two types: α and β, and they can activate eNOS activity of endothelial cells, inhibit VSMC proliferation, reduce pathological myocardial hypertrophy and regulate mitochondrial gene expression (Knowlton and Lee, 2012). Estrogen can inhibit the apoptosis and necrosis of cardiomyocytes and endothelial cells, reduce pathological cardiac remodeling, and play a certain anti-inflammatory role. In addition, estrogen can also upregulate antioxidant genes, increase eNOS activity and reduce the production of superoxide. These may provide new support for the internal mechanism of resveratrol inhibiting cardiac remodeling, but some specific mechanisms still need to be further explored.

### 2.9 Cardiomyocytes’ proliferation

The heart has always been considered as an organ of terminal differentiation, in which myocardial cells are considered not to have the ability of regeneration, re differentiation and self-repair. However, the discovery of cardiac progenitor cells (CPCs) may reverse the previous understanding (Kattman et al., 2006; Wu et al., 2006). They are resident stem cells in the heart and have the ability of self-renewal, proliferation and differentiation. They provide the possibility for the treatment of myocardial infarction, heart failure and other diseases, such as rescuing damaged myocardium, providing heart transplantation, and finally restoring cardiac function (Cai et al., 2003; Henning, 2011). CPCs have the potential to differentiate into various types of cardiomyocytes and can promote angiogenesis (Beltrami et al., 2003; Yang et al., 2008; Tallini et al., 2009). However, there are still many unknown parts. For example, the transplantation efficiency of CPCs is low and the cell viability is poor. In addition, it may be affected by *in vivo* inflammation, original pathological changes of the heart, internal environment, telomerase activity and so on (Vanhoutte et al., 2006; Dobaczewski et al., 2010b; Deddens et al., 2017), which greatly reduces the efficiency of this scheme. In addition, there may even be new side effects, such as arrhythmia, teratoma, etc (Le et al., 2017). At present, researchers hope to improve the success rate of CPCs transplantation by using resveratrol. It has been found that resveratrol can prolong the process of myogenic differentiation and control the cell cycle of cardiomyoblasts (Abe et al., 2018). Thanks for the MITO-Porter system, mitochondrion was used to deliver resveratrol in order to activate CPCs, which could upregulate mitochondrial enzyme activity, enhance mitochondrial membrane potential, improve the process of mitochondrial biogenesis, promote the reactivation of cell cycle of CPCs and help improving the efficiency of CPCs transplantation (Abe et al., 2018). In addition, resveratrol can play an antioxidant role, reduce the level of oxidative stress, ensure the survival of cardiac stem cells, complete differentiation and proliferation, and then improve cardiac function. In the future, we believed that the further research on resveratrol could make great contribution to cardiac rehabilitation, improving left ventricular ejection function.

At the end of this review, we sorted out the molecular mechanism of resveratrol in different cardiac pathological remodeling phenotypes of independent cardiovascular diseases in the past year, as shown in Table 2. Inclusion criteria: 1) cardiovascular diseases included: hypertension, coronary heart disease, cardiomyopathy, heart valve disease and heart failure; 2) research published from 1 January 2021 to 7 June 2022; 3) search scope: PubMed. Exclusion criteria: 1) studies of other drug-induced diseases, such as adriamycin induced cardiotoxicity, dioxin induced hypertension, etc; 2) studies where resveratrol is not the main study medication; 3) review; 4) studies aiming at diseases other than the above-mentioned cardiovascular diseases.

**TABLE 2:** Molecular mechanism of resveratrol in different cardiac remodeling phenotypes of independent cardiovascular disease in recent 1 year.

No.	Year	Disease	Drugs	Models	Effects	Mechanism	Phenotype	Reference
1	2021	Hypertension	ADMA; TMAO; RSV (50 mg/L)	Pregnant SD rats (ADMA and/or TMAO)	↑ Abundance of the butyrate-producing genera Lachnospiraceae, Ruminococcaceae, the Cyanobiaceae and Erysipelotrichaceae families; ↑ Fecal butyrate levels; ↓ Angiotensinogen; ↓ Renin; ↓ Prorenin receptor; ↓ ACE; ↓ AT1R; ↑ ACE2; ↑ AT2R; ↑ MAS	Regulating gut microbiota and their metabolites, RAS, NO pathway	Gut microbiome	Hsu et al. (2021)
2	2021	Hypertension	RSV (10 mg/kg/day); L-NAME	SHR; MHR	↓ BP; ↓ Lipid peroxidation; ↑ Oxidative status; ↑ NO; Ameliorating myocardial morphological changes	Antioxidant, anti-inflammatory and anti-apoptotic properties	Hypertrophy; Apoptosis	Grujić-Milanović et al. (2021)
3	2021	Hypertension	RSV (15 mg/kg)	Male Wistar rats (deoxycorticosterone-acetate + salt administration)	↓ SBP; Improving adrenergic and cholinergic responses of the right atrium and left papillary muscles; ↓ TAC; ↑ Expression of antioxidant genes; ↑ PINK1; ↓ NLRP3 inflammasome activation; ↑ Caspase-3, Bax, and Bcl-2; ↓ Phosphorylation of stress-related mitogenic proteins p38 and JNK	Oxidative stress; ER stress; Mitophagy; NLRP3 inflammasome-mediated inflammation; mitogenic activation	Cellular stress responses; Apoptosis	Bal et al. (2022)
4	2021	CHD	RSV (25 mg/kg/d); LY294002	SD rats (CME)	↑ Cardiac dysfunction; ↓ The serum level of myocardial injury biomarkers; ↓Myocardial microinfarct size; ↓Cardiomyocyte apoptotic index; ↓ Proteins and mRNAs associated with the pro-apoptosis; ↑Proteins and mRNAs associated with the anti-apoptosis	Regulating PI3K/Akt/GSK-3β cascade pathway	Apoptosis	Li et al. (2021)
5	2021	CHD; I/R injury; Cardiac arrhythmias	RSV (1 mg/kg/day); Grape juice; Red wine	Male Wistar rats (I/R)	↓ Incidence of AVB; ↓ Incidence of LET; ↓ Na^+^ channels; ↓Transient and sustained K^+^ currents	Blocking cardiac L-type Cav; ↑ Cardiac refractory period	Anti-arrhythmic action	Menezes-Rodrigues et al. (2021)
6	2021	CHD; I/R injury; Cardiac arrhythmias	RSV (2.5 mg/kg/d)	Male Wistar rats (I/R)	↓ Mas receptor mRNA level; ↓ QT-interval duration; ↓ Infarct size; ↓ Incidence of ischemia-induced arrhythmia; ↑ The cardiac level of Ang (1–7)	Enhancing Ang (1–7)/MasR axis	Anti-ischemic effect	Soltan et al. (2021)
7	2022	CHD; I/R injury	RSV (50 mg/kg)	H9c2 cells (OGD/R model); I/R rats	↓ Oxidative stress; ↓ Fe^2+^ content; ↓ Ferroptosis; ↓ TfR1 expression; ↑ FTH1; ↑ GPX4	Inhibiting ferroptosis by the regulation of USP19-Beclin1 autophagy	Ferroptosis; Autophagy	Li et al. (2022)
8	2021	CHD; I/R injury	RSV (20 mg/kg)	I/R SD rats	↓ TNF-α; ↓ RIP1; ↓ RIP3; ↓ p-MLKL/MLKL; ↑ Cell viability; ↓ Necroptosis; ↓ The enhanced effect of TNF-α on necroptosis in myocardial H/R-injured cells	Inhibiting TNF-α/RIP1/RIP3/MLKL signaling pathway	Necroptosis	Hu et al. (2021)
9	2021	CHD; Myocardial ischemic injury	RSV (2 mg/kg/day)	Rat H9C2 cardiac myoblasts (5% CO2 and 95% air at 37 °C); AMI male C57BL/6 mice (LAD ligation)	↓ ROS; ↑ SOD; ↑ GSH; ↑GPX; ↓ p-IKK; ↓ p-NF-κB p65; ↓ IL-1β; ↓ IL-6; ↓ NGF; ↓ IGF-1	Inhibiting oxidative stress and inflammatory responses	Hypoxia-induced apoptosis	He et al. (2021)
10	2022	DCM	RSV (10 mg/kg); RSV (50 mg/kg)	DCM rats	↓ Heart weight; ↓ Heart weight/body weight ratio; ↓ LVEDD; ↓ LVESD; ↓ Myocardial fibrosis; ↓ Col I; ↓ Col III; ↑ SIRT1 mRNA; ↓ Ac-Smad3	Regulating SIRT1/Smad3 deacetylation pathway	Myocardial fibrosis	Chen et al. (2022)
11	2021	HFpEF	RES (10 mg/kg/day); d-aldosterone	C57BL/6 mice (uninephrectomy surgery and d-aldosterone infusion)	Reversing HFpEF-induced cardiac remodeling; ↓ Smad3 acetylation; ↓ Smad3 transcriptional activity; The protective effect of RSV on TGF-β-induced cardiac fibroblast-myofibroblast transformation in CFs	Decreasing Smad3 acetylation and transcriptional activity via activating SIRT1	Cardiac remodeling	Zhang et al. (2021)
12	2021	HF induced by MI	RSV (2.5 mg/kg/day); sacubitril/valsartan; valsartan alone	MI-induced male SD rats (permanent ligation of LAD)	↓ LV dilatation; ↑ LVEF; ↓ MDA; ↓ Myocardial tissue oxidative stress; ↓ Inflammation; ↓ Fibrosis; ↓ BNP; Preventing the increase of TNF-α	Reducing cardiac oxidative stress, inflammation and fibrosis	Cardiac remodeling	Raj et al. (2021b)

ADMA, asymmetric dimethylarginine; TMAO, trimethylamine-N-oxide; RSV, resveratrol; SD rats, Sprague-Dawley rats; RAS, the renin-angiotensin system; NO, nitric oxide; ACE, angiotensin converting enzyme; AT1R, angiotensin II type 1 receptor; AT2R, angiotensin II type 2 receptor; MAS, angiotensin (1-7) receptor MAS; SHR, spontaneously hypertensive rats; MHR, malignantly hypertensive rats; BP, blood pressure; L-NAME, a nonselective inhibitor of NO synthase enzyme; SBP, systolic blood pressure; PINK1, mitophagic marker PTEN-induced putative kinase 1; PTEN, phosphatase and tensin homolog; NLRP3, nucleotide-binding oligomerization domain, leucine-rich repeat and pyrin domain- containing 3; Bax, BCL2-Associated X; Bcl-2, B-cell lymphoma-2; JNK, c-Jun N-terminal protein kainse; PI3K, phosphoinositide 3-kinase; Akt, protein kinase B; GSK-3β, glycogen synthase kinase-3β; ER, endoplasmic reticulum; SIRT1, sirtuin 1; CHD, coronary heart disease; CME, coronary microembolization; AVB, atrioventricular block; LET, lethality; MasR, Mas receptor; Mas, mitochondrial assembly; Ang, angiotensin; OGD/R, oxygen-glucose deprivation/reoxygenation; TfR1, transferrin receptor 1; GPX4, glutathione peroxidase 4; FTH1, ferritin heavy chain 1; USP19, ubiquity specific peptidase 19; I/R, ischemia/reperfusion; H/R, hypoxia/reoxygenation; TNF-α, tumor necrosis factor-alpha; RIP1, receptor-interacting protein kinase 1; MLKL, mixed-lineage kinase domain-like; AMI, acute myocardial infarction; ROS, reactive oxygen species; SOD, superoxide dismutase; GSH, glutathione; p-IKK, phosphorylated (p-)IκB kinase; p-NF-κB p65, p-nuclear factor (NF)-κB p65; IL-1β, interleukin-1β; NGF, nerve growth factor; IGF-1, insulin-like growth factor-1; DCM, dilated cardiomyopathy; LVEDD, left ventricular end diastolic diameter; LVESD, left ventricular end systolic diameter; Col I, collagen type I; Ac-Smad3, Acetylation of Smad3; HFpEF, heart failure with preserved ejection fraction; HF, heart failure; MI, myocardial infarction; TGF-β, transforming growth factor β1; LV, left ventricular; EF, ejection fraction; BNP, brain natriuretic peptide.

## 3 Conclusion

Resveratrol plays an important role in antioxidation, anti-inflammatory effect, regulating immune cells and improving vascular function, improving neurohumoral regulation, improving cardiomyocytes’ proliferation, promoting autophagy, inhibiting myocardial fibrosis and its impact on mitochondrial degradation and lipid metabolism, so as to achieve the protective purpose of maintaining the normal function of myocardial cells. Therefore, resveratrol has the advantage of inhibiting cardiac remodeling (Gutlapalli et al., 2020). This review concludes the research of resveratrol in cardiac remodeling, in order to provide support for its research in the prevention and treatment of cardiac remodeling related diseases.
